# Mechanism of *Impatiens glandulifera* Royle Allelopathy to *Sinapis alba* L. and *Raphanus sativus* L. Germination Is Through Oxidative Stress

**DOI:** 10.3390/plants14182901

**Published:** 2025-09-18

**Authors:** Ana-Marija Domijan, Maja Bival Štefan, Ivan Duka, Tihana Marić, Maja Friščić, Željan Maleš, Božena Mitić, Dario Hruševar

**Affiliations:** 1 Faculty of Pharmacy and Biochemistry, University of Zagreb, A. Kovačića 1, 10000 Zagreb, Croatia; mbival@pharma.hr (M.B.Š.); iduka@pharma.hr (I.D.); tmaric@pharma.hr (T.M.); mfriscic@pharma.hr (M.F.); zmales@pharma.hr (Ž.M.); 2 Faculty of Science, University of Zagreb, Marulićev trg 9A, 10000 Zagreb, Croatia; bozena.mitic@biol.pmf.hr (B.M.); dario.hrusevar@biol.pmf.hr (D.H.)

**Keywords:** allelopathy, *Impatiens glandulifera*, germination, oxidative stress, naphthoquinones

## Abstract

*Impatiens glandulifera* Royle (*I. glandulifera*) in the EU presents a threat to the ecosystem, and is thus on the list of invasive alien species. The aim of this study was to clarify if an allelopathic effect of *I. glandulifera* involves the activation of oxidative stress in target plants. First, *I. glandulifera* leaf extract was prepared and levels of 2-hydroxy-1,4-naphtoquinone (2-HNQ) and 2-methoxy-1,4-naphthoquinone (2-MNQ), as main allelochemicals of *I. glandulifera*, determined by HPLC. Next, seeds of white mustard (*Sinapis alba*) or radish (*Raphanus sativus*) were exposed to the extract or to 2-MNQ (in the concentration range of 1–30 µg/mL) for 3 days and parameters of germination and oxidative stress were assessed. Both the leaf extract and 2-MNQ inhibited germination of white mustard and radish; however, the effect of 2-MNQ was more pronounced. Furthermore, the extract and 2-MNQ induced activation of antioxidative defense and caused oxidative damage to lipids and proteins in white mustard and radish seedlings. It was observed that radish seedlings were less susceptible to negative effect of *I. glandulifera* extract and 2-MNQ. This study’s obtained results demonstrated that 2-MNQ is the main allelochemical of *I. glandulifera* and that the mechanism by which *I. glandulifera* and 2-MNQ induce negative effects to target plants involves oxidative stress. In addition, species-dependent susceptibility to *I. glandulifera* and 2-MNQ was shown.

## 1. Introduction

*I. glandulifera* (Balsaminaceae) is annual plant, tall (1–4 m of height) with large, red–violet flowers [1,2]. It is native to western Himalaya, from where it was introduced to Europe and North America as an ornamental garden plant in 1839 [3,4]. *I. glandulifera* is distributed through whole Europe and become naturalized mainly by rivers and in forests [3,5].

In most European countries, *I. glandulifera* is extremely invasive, affecting negatively ecosystem. *I. glandulifera* affects native plants by changing soil characteristics, microclimate, and competing for pollinators [3]. Because of its height (it reaches up to 4 m height), *I. glandulifera* is strong competitor to native herbaceous species. *I. glandulifera* has extremely high seed production, and seeds are efficiently dispersed [6]. Due to its invasiveness, in 2017, the EU placed *I. glandulifera* on the list of invasive alien species [1,5].

Phytochemical studies revealed that *I. glandulifera* has high content of naphthoquinones (NQs), predominantly 2-hydroxy-1,4-naphthoquinone (2-HNQ) and 2-methoxy-1,4-naphthoquinone (2-MNQ) [7,8]. Our previous study confirmed the presence of 2-MNQ—but not 2-HNQ—in the leaves and flowers of *I. glandulifera* collected in Croatia [9]. It was suggested that 2-MNQ is the main metabolite produced by *I. glandulifera* that has an allelopathic effect on co-occurring plants [1,5]. Ruckli et al. [4] found that 2-MNQ can be rinsed by rain from *I. glandulifera* leaves and exuded from *I. glandulifera* roots, thus reaching soil and co-occurring plants.

In plants, in response to biotic and abiotic stresses, overproduction of reactive oxygen species occur [10,11]. The accumulation of reactive oxygen species can lead to damage of cells’ macromolecules including lipids and proteins, activate synthesis of antioxidant enzymes, such as superoxide dismutase (SOD), and induce production of antioxidant non-enzymatic substances including glutathione (GSH) and polyphenols [10,11]. In plants, biosynthesis of polyphenols is usually increased to endure different biotic and abiotic stresses [11].

Up to now, physiological changes induced by *I. glandulifera* per se or its metabolite 2-MNQ to target plants have not been clarified. Therefore, the aim of this study was to investigate oxidative stress as possible physiological mechanism of inhibition (plant–plant interaction) of *I. glandulifera* to target plants. In the first step of the study, leaves of *I. glandulifera* were collected, extract prepared and levels of 2-MNQ and 2-HNQ in the extract were quantified. Seedlings are particularly sensitive to environmental cues [12,13]. Therefore, in the next step, seeds of white mustard *Sinapis alba* L. (*S. alba*) and radish *Raphanus sativus* L. (*R. sativus*) were exposed to *I. glandulifera* leaf extract and germination, oxidative stress parameters and level of polyphenols were determined. In order to confirm that the main inhibitory metabolite of *I. glandulifera* is 2-MNQ, in the next step, seeds of white mustard and radish were exposed to 2-MNQ and germination, oxidative stress parameters and level of polyphenols in seedlings were monitored.

## 2. Results

### 2.1. Level of NQs in Plant Extract

For this study, leaves of *I. glandulifera* were collected and extract of air-dried plant material was prepared by decoction in methanol. Level of NQs was quantified by HPLC. The method was previously validated [9] and was confirmed that method is linear, precise and sensitive and therefore suitable for determination of 2-MNQ and 2-HNQ in plant material. Prepared extract was analyzed using HPLC in triplicate. In extract of *I. glandulifera* leaves only 2-MNQ was detected (in the concentration: 20.6 ± 1.4 µg/mL), while concentration of 2-HNQ was under the detection limit of the method (0.1 µg/mL).

### 2.2. Germination

#### 2.2.1. Effect of *I. glandulifera* Leaf Extract on Germination of White Mustard and Radish Seeds

**Figure 1:**
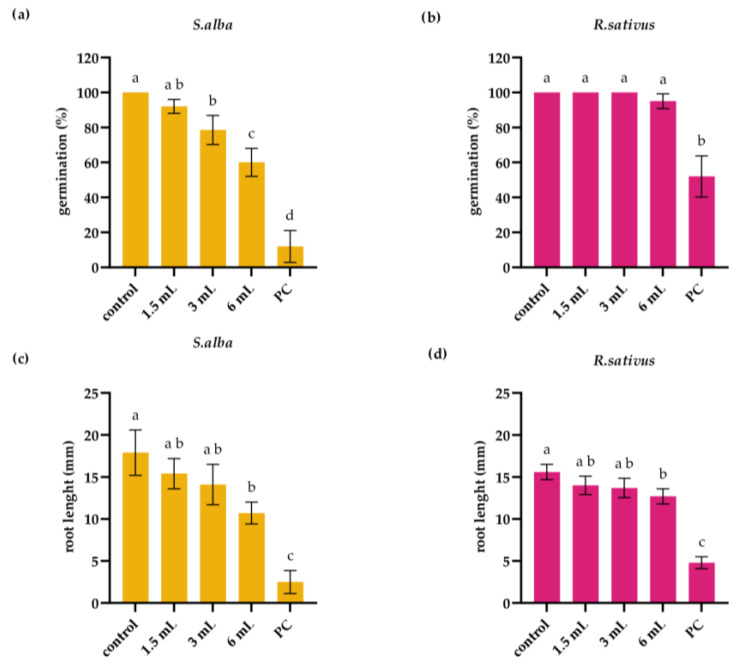
Germination (**a**,**b**) and root length (**c**,**d**) of white mustard (*S. alba*) and radish (*R. sativus*) following 3 days of exposure to *I. glandulifera* leaf extract. PC—positive control (0.02 M CuSO_4_). Different letters indicate significantly different values at *p* < 0.05.

Extract of *I. glandulifera* leaf reduced the germination rate of white mustard seeds, while it had no effect on the germination rate of radish seeds (Figure 1a,b). Three days of exposure to the extract at levels 3 mL and 6 mL significantly reduced germination in white mustard seeds (to 78.6 ± 8.3% and to 60 ± 8%, respectively) compared to a negative control (100%) (*p* < 0.05; Figure 1a).

On the other hand, *I. glandulifera* leaf extract reduced root elongation of both, white mustard and radish seedlings (Figure 1c,d). Compared to the root length of the control white mustard seedlings (17.9 ± 2.7 mm) exposure to the extract at level 6 mL reduced root length to 10.7 ± 1.3 mm (*p* < 0.05; Figure 1c). Similarly, compared to root length of control radish seedlings (15.6 ± 0.9 mm) the extract at level 6 mL reduced root length to 12.7 ± 1.1 mm (*p* < 0.05; Figure 1d).

Regarding fresh weight, the extract significantly affected only white mustard fresh weight (Table 1). Fresh weight of control white mustard seedlings was 0.61 ± 0.03 g, while the extract at level 1.5 mL reduced fresh weight of white mustard seedlings to 0.42 ± 0.04 g (*p* < 0.05; Table 1). Upon exposure to *I. glandulifera* extract, the fresh weight of radish seedlings was slightly reduced, but this reduction was not significant (*p* > 0.05; Table 1). The correlation among germination parameters of white mustard seeds and among germination parameters of radish seeds following exposure to *I. glandulifera* leaf extract was observed; correlation coefficients, r, ranged from 0.760 to 0.923 (Supplementary Tables S1 and S2).

#### 2.2.2. Effect of 2-MNQ on Germination of White Mustard and Radish Seeds

**Figure 2:**
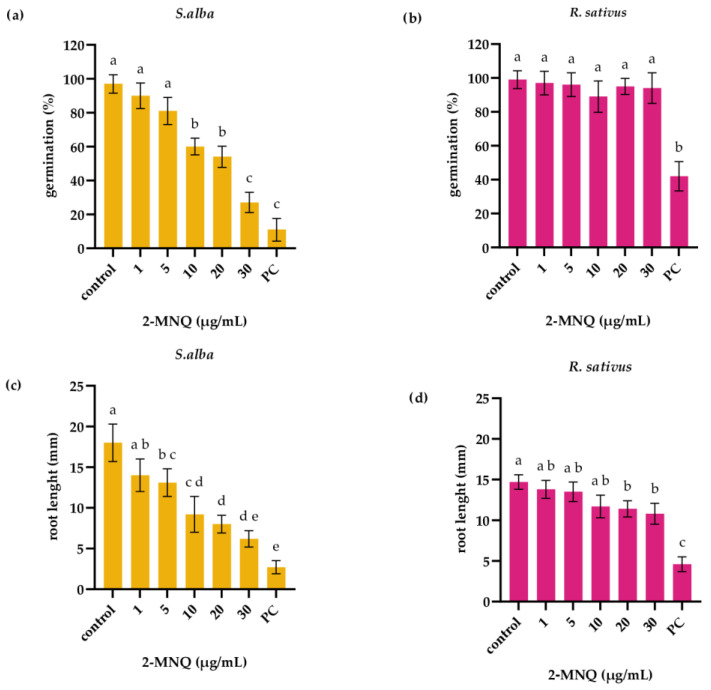
Germination rate (**a**,**b**) and root length (**c**,**d**) of white mustard (*S. alba*) and radish (*R. sativus*) following 3 days of exposure to 2-methoxy-1,4-naphtoquinone (2-MNQ). PC—positive control (0.02 M CuSO_4_). Different letters indicate significantly different values at *p* < 0.05.

Next, the effect of 2-MNQ (in the concentration range: 1–30 µg/mL) on germination of white mustard and radish seeds was tested. Three days of exposure to 2-MNQ at a concentration of 10 µg/mL significantly reduced the germination rate of white mustard seeds (60 ± 4.9% compared to control 97 ± 5.4%; *p* < 0.05; Figure 2a) while the exposure to 2-MNQ, even at the highest applied concentration (30 µg/mL), had no effect on germination rate of radish seeds (94 ± 9% compared to control 99 ± 5.2%; Figure 2b).

The root length of both white mustard and radish seedlings was reduced upon exposure to 2-MNQ (Figure 2c,d); however, reduction in the root length of white mustard seedlings was observed at lower 2-MNQ concentration (5 µg/mL), compared to radish (20 µg/mL). Root length of control seedlings of white mustard was 18 ± 2.3 mm while root length of white mustard seedlings exposed to 2-MNQ at concentration 5 µg/mL was 13.1 ± 1.7 mm (*p* < 0.05; Figure 2c). Radish seedlings exposed to 2-MNQ at concentration 20 µg/mL had a root length of 11.4 ± 1.1 mm that was significantly lower than the root length of control seedlings (14.7 ± 0.9 mm; *p* < 0.05; Figure 2d).

Three days of exposure to 2-MNQ reduced fresh weight of white mustard seedlings. The fresh weight of white mustard seedlings exposed to 2-MNQ at concentration 10 µg/mL was 0.28 ± 0.07 g, which was significantly lower than the fresh weight of control seedlings (0.34 ± 0.04 g; *p* < 0.05; Table 2). However, no significant reduction in the fresh weight of radish seedlings was observed upon exposure to 2-MNQ (Table 2). Correlation coefficients, r, among germination parameters of white mustard seeds and germination parameters of radish seeds following exposure to 2-MNQ, ranged from 0.730 to 0.884, showing a correlation among germination parameters (Supplementary Tables S3 and S4).

### 2.3. Oxidative Stress Parameters

#### 2.3.1. Effect of *I. glandulifera* Leaf Extract on Oxidative Stress Parameters of White Mustard and Radish Seedlings

**Figure 3:**
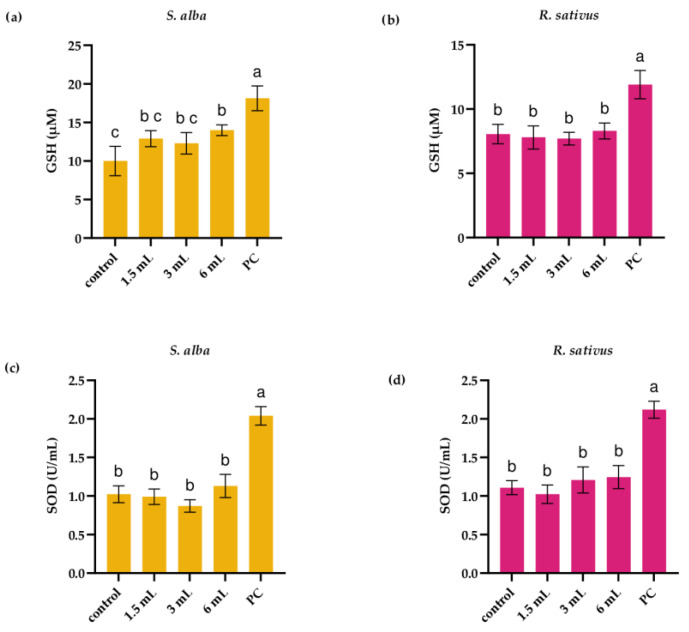
Level of glutathione (GSH) (**a**,**b**) and catalytical activity of superoxide dismutase (SOD) (**c**,**d**) in seedlings of white mustard (*S. alba*) and radish (*R. sativus*) following 3 days of exposure to *I. glandulifera* leaf extract. PC—positive control (0.02 M CuSO_4_). Different letters indicate significantly different values at *p* < 0.05.

To estimate the antioxidative status of white mustard and radish seedlings following 3 days of exposure to extract of *I. glandulifera* leaves, the level of GSH and catalytical activity of SOD were assessed. In white mustard seedlings, exposure to the extract increased the GSH level. Exposure to the extract at level of 6 mL increased level of GSH to 13.4 ± 0.7 µM compared to control seedlings (10.3 ± 1.9 µM; *p* < 0.05; Figure 3a). Exposure to *I. glandulifera* leaf extract had no effect on level of GSH in radish seedlings (Figure 3b). Three days of exposure to *I. glandulifera* leaf extract had no significant effect on catalytical activity of SOD in white mustard and radish seedlings (Figure 3c,d).

**Figure 4:**
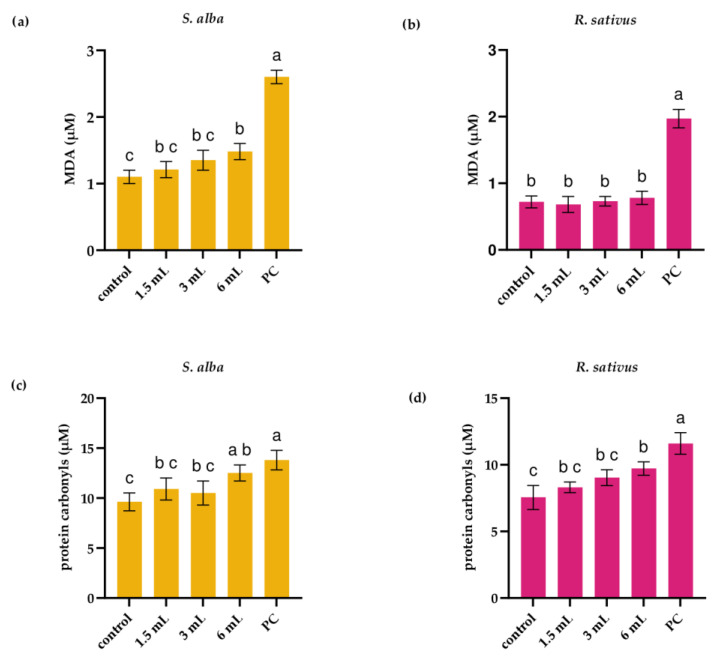
Level of malondialdehyde (MDA) (**a**,**b**) and protein carbonyls (**c**,**d**) in seedlings of white mustard (*S. alba*) and radish (*R. sativus*) following 3 days of exposure to *I. glandulifera* leaf extract. PC—positive control (0.02 M CuSO_4_). Different letters indicate significantly different values at *p* < 0.05.

As markers of oxidative damage after 3 days of exposure to *I. glandulifera* leaf extract in white mustard and radish seedlings levels of MDA and protein carbonyls were assessed (Figure 4). In white mustard seedlings increase in both MDA and protein carbonyls was observed. MDA level in white mustard seedlings exposed to the extract at level 6 mL was 1.48 ± 0.12 µM that was significantly higher than MDA level in control seedlings (1.1 ± 0.1 µM; *p* < 0.05; Figure 4a). In comparison to control white mustard seedlings, in which the level of protein carbonyls was 9.62 ± 0.9 µM, in white mustard seedlings exposed to the extract at level 6 mL, the level of protein carbonyls was 12.3 ± 0.8 µM (*p* < 0.05; Figure 4c). In radish seedlings, an increase in MDA level was not observed (Figure 4b). However, the extract at level 6 mL increased level of protein carbonyls in radish seedlings (9.72 ± 0.5 µM) compared to control seedlings (7.55 ± 0.9 µM, *p* < 0.05; Figure 4d).

In white mustard seedlings exposed to the *I. glandulifera* extract, a strong negative correlation among germination rate and oxidative stress parameters was recorded (Supplementary Table S1). Correlation coefficient, r, between germination rate and GSH was −0.926, between germination rate and SOD was −0.902, between germination rate and MDA was −0.968 and between germination rate and protein carbonyls was −0.904. Similarly, in radish seedlings, strong negative correlation among germination rate and oxidative stress parameters was observed with correlation coefficients ranging from r = −0.992 (between germination rate and MDA) to r = −0.855 (between germination rate and protein carbonyls) (Supplementary Table S2). Among the oxidative stress parameters of white mustard and among the oxidative stress parameters of radish, a strong positive correlation was observed.

#### 2.3.2. Effect of 2-MNQ on Oxidative Stress Parameters of White Mustard and Radish Seedlings

**Figure 5:**
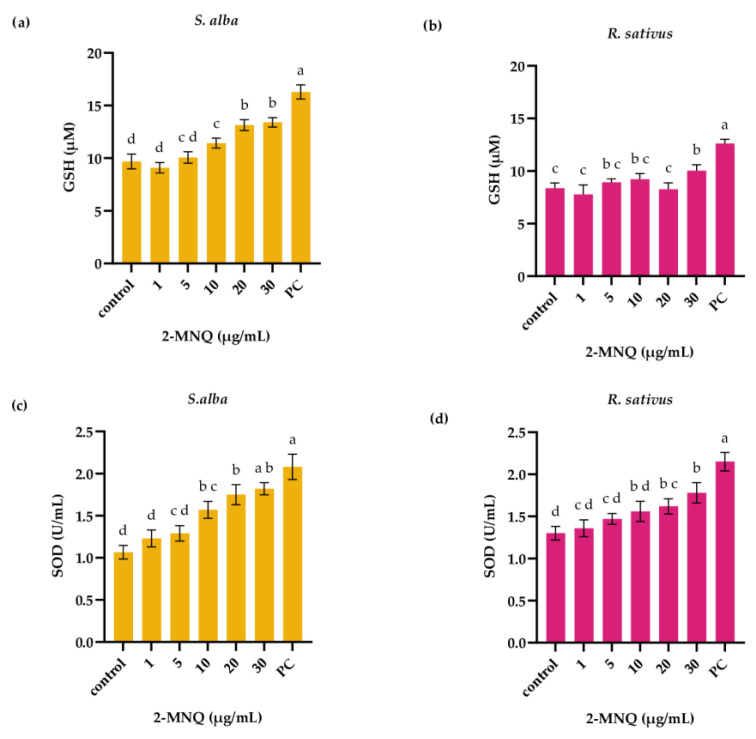
Level of glutathione (GSH) (**a**,**b**) and catalytical activity of SOD (**c**,**d**) in seedlings of white mustard (*S. alba*) and radish (*R. sativus*) following 3 days of exposure to 2-methoxy-1,4-naphtoquinone (2-MNQ). PC—positive control (0.02 M CuSO_4_). Different letters indicate significantly different values at *p* < 0.05.

In the next step, the impact of 2-MNQ (1–30 µg/mL) on antioxidative status in white mustard and radish seedlings was investigated. Three days of exposure to 2-MNQ increased level of GSH and catalytical activity of SOD in white mustard seedlings. Compared to control white mustard seedlings (9.69 ± 0.7 µM) level of GSH in white mustard seedlings exposed to 2-MNQ at concentration 10 µg/mL was 11.43 ± 0.47 µM (*p* < 0.05; Figure 5a). Catalytical activity of SOD in control white mustard seedlings was 1.07 ± 0.08 U/mL, while SOD activity in white mustard seedlings exposed to 2-MNQ (10 µg/mL) was 1.57 ± 0.1 U/mL (*p* < 0.05; Figure 5c). Similarly, exposure to 2-MNQ increased level of GSH and activity of SOD in radish seedlings, but only at higher applied concentrations; the level of GSH was increased following exposure to 2-MNQ at concentration 30 µg/mL, while SOD activity was increased at 2-MNQ concentration 20 µg/mL (Figure 5b,d).

**Figure 6:**
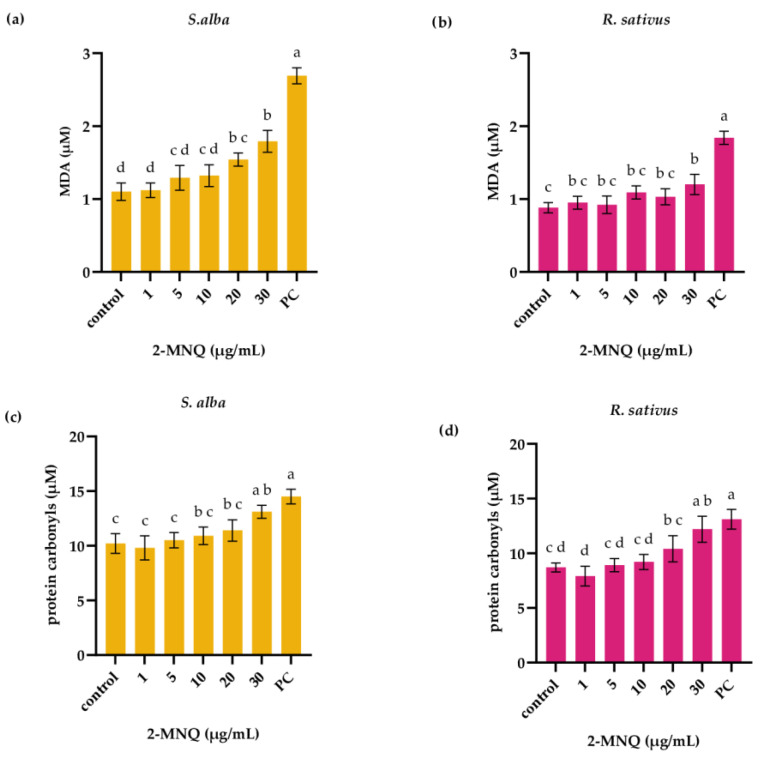
Level of malondialdehyde (MDA) (**a**,**b**) and protein carbonyls (**c**,**d**) in seedlings of white mustard (*S. alba*) and radish (*R. sativus*) following 3 days of exposure to 2-methoxy-1,4-naphtoquinone (2-MNQ). PC—positive control (0.02 M CuSO_4_). Different letters indicate significantly different values at *p* < 0.05.

In white mustard seedlings, 3 days of exposure to 2-MNQ increased the level of both parameters of oxidative damage, MDA and protein carbonyls (Figure 6a,c). 2-MNQ in concentration 20 µg/mL increased the MDA level to 1.54 ± 0.09 µM compared to control seedlings (1.1 ± 0.12 µM, *p* < 0.05; Figure 6a). The level of protein carbonyls in white mustard seedlings was significantly higher only following exposure to the highest 2-MNQ concentration (30 µg/mL). In radish seedlings, 2-MNQ only at the highest applied concentration (30 µg/mL) affected level of MDA and protein carbonyls. In control radish seedlings, the level of MDA was 0.88 ± 0.07 µM, while in radish seedlings exposed to 2-MNQ with a concentration of 30 µg/mL, the level of MDA was 1.22 ± 0.14 µM (*p* < 0.05; Figure 6b). The level of protein carbonyls in control radish seedlings was 8.7 ± 0.4 µM, while in radish seedlings exposed to 2-MNQ in concentration of 30 µg/mL level of protein carbonyls was 12.2 ± 1.2 µM (*p* < 0.05; Figure 6d). Following exposure to 2-MNQ, a strong negative correlation among germination parameters and oxidative stress parameters of white mustard and of radish were observed (Supplementary Tables S3 and S4). Correlation coefficients ranged from r = −0.944 (between germination rate and SOD activity; white mustard seedlings) to r = −0.714 (between fresh weight and protein carbonyls; radish seedlings). A strong positive correlation was observed among oxidative stress parameters of white mustard and among oxidative stress parameters of radish (Supplementary Tables S3 and S4).

### 2.4. Total Polyphenols

#### 2.4.1. Effect of *I. glandulifera* Leaf Extract on Level of Polyphenols in White Mustard and Radish Seedlings

Level of total polyphenols (TP) in white mustard seedlings following 3 days of exposure to *I. glandulifera* extract at level 3 mL (397.3 ± 12.7 µg/mL) was higher compared to control seedlings (342.3 ± 21.9; *p* < 0.05; Table 3). The extract only at level 6 mL increased level of TP in radish seedlings compared to control seedlings (424.4 ± 24.0 µg/mL vs. 373.9 ± 21.9 µg/mL; *p* < 0.05; Table 3). In white mustard and radish seedlings exposed to *I. glandulifera* leaf extract, a strong negative correlation among level of TP and germination parameters and a strong positive correlation among level of TP and oxidative stress parameters were recorded (Supplementary Tables S1 and S2). Correlation coefficients ranged from r = −0.968 (between germination rate and TP in white mustard seedlings) to r = 0.835 (between GSH and TP in radish seedlings).

#### 2.4.2. Effect of 2-MNQ on Level of Polyphenols in White Mustard and Radish Seedlings

In both white mustard seedlings and radish seedlings, 2-MNQ increased the level of TP (Table 4). The level of TP in white mustard seedlings exposed to 2-MNQ at a concentration of 5 µg/mL was 396.7 ± 10.8 µg/mL, which was higher than level of TP in control seedlings (351 ± 15.7 µg/mL; *p* < 0.05, Table 4). In control radish seedlings, the level of TP was 372 ± 18.0 µg/mL, while in radish seedlings exposed to 2-MNQ at a concentration of 10 µg/mL, the level of TP was 442.3 ± 9.3 µg/mL (*p* < 0.05; Table 4). In white mustard and in radish seedlings exposed to 2-MNQ, a strong negative correlation among levels of TP and germination parameters and a strong positive correlation among levels of TP and oxidative stress parameters were observed (Supplementary Tables S3 and S4).

## 3. Discussion

In Europe, *I. glandulifera* is an invasive species, thus presenting a serious threat to the ecosystem in the EU including in Croatia. In 2017, the EU placed *I. glandulifera* on the list of invasive alien species [1,5]. Species from *Impatiens* genera produce high amounts of NQs, predominantly 2-HNQ and 2-MNQ [7,8]. In our previous study, it was found that *I. glandulifera* collected in Croatia contains 2-MNQ as a major NQ [9]. This was also confirmed in the current study.

Studies indicate that the inhibitory effect of *I. glandulifera* on co-occurring plants is accomplished via 2-MNQ [3,4]. Ruckli et al. [4] found that 2-MNQ is released from *I. glandulifera* by exuding from its roots and leaching from its leaves by rain. In their study, they detected 2-MNQ in soil and in rainwater rinsed from *I. glandulifera* leaves. This could be the way in which *I. glandulifera* modifies the chemical composition of soil and can affect native organisms in their habitat.

In this study, the inhibitory effect and mechanism of inhibitory effect on target plants of *I. glandulifera* leaf extract were studied by examining its impact on germination and parameters of oxidative stress of white mustard and radish seedlings. In addition, the impact of 2-MNQ, as the main inhibitory phytochemical (allelochemical) of *I. glandulifera*, on germination and oxidative stress of white mustard and radish seedlings were investigated. Plant extract is commonly used to investigate allelopathy in experimental conditions [14,15,16,17,18,19,20,21]. Leaves are considered as main source of allelochemicals, and therefore, it is suggested to employ leaf extract to study allelopathic potential [20]. Mechanism of allelopathy is tested on plant models that willingly germinate [14,15,16,17,18,19]. Therefore, for this study white mustard and radish seeds were selected since are commonly used for phytotoxicity studies, readily germinate [13] and are native plants to Croatia.

Results of this study indicate that extract of *I. glandulifera* leaves affects germination of both, white mustard and radish seeds. Extract of *I. glandulifera* leaves inhibited growth of white mustard seedlings which was observed by dose-dependent reduction in fresh weight, root elongation and germination rate. The extract also affected the growth of radish seedlings; however, only a reduction in the elongation of radish seedlings roots was recorded, while the extract had no impact on radish seedlings’ fresh weight and germination rate. This implies that white mustard seedlings, in comparison to radish seedlings, are more susceptible to inhibitory effect of *I. glandulifera* leaf extract. Several other studies demonstrated that *I. glandulifera* extract inhibits seeds germination. Vrchotova et al. [2] observed that aqueous and methanol *I. glandulifera* extracts reduced germination and radicle elongation of *Leucosinapis alba* and *Brassica napus* seeds. Aqueous shoot and root extracts *of I. glandulifera* inhibited germination of native forest plants *Hieracium murorum* and *Scrophularia nodosa* and the inhibition of seeds germination correlated with 2-MNQ concentration in the extracts [4]. Bieberich et al. [1] exposed germinated seeds of *Filipendula ulmaria* (*F. ulmaria*), *Urtica dioica* (*U. dioica*), *Salix fragilis* (*S. fragilis*), *Lepidium sativum* (*L. sativum*), and *Geum urbanum* (*G. urbanum*) (species co-occurring with *I. glandulifera*) to leaf material of *I. glandulifera* seedlings. *I. glandulifera* leaf material reduced root length of *G. urbanum*, *U. dioica*, *S. fragilis* and *L. sativum* but only reduced seedling biomass of *U. dioica*, thus the authors concluded that species responded differently to *I. glandulifera* leaf material, *F. ulmaria* being the least affected and *U. dioica* the most. Thus, our observation on different species response to *I. glandulifera* is in line with study of Bieberich et al. [1].

To confirm that inhibitory effect of *I. glandulifera* leaf extract is through 2-MNQ, impact of 2-MNQ on germination of white mustard and radish seeds was tested. 2-MNQ affected the germination of white mustard and radish seeds in a similar manner as *I. glandulifera* leaf extract. The obtained results demonstrated that 2-MNQ can inhibit the growth of white mustard and radish seedlings, and that radish seedlings are less susceptible to negative effect of 2-MNQ. It is important to stress that *I. glandulifera* leaf extract reduced the root elongation of white mustard and radish seedlings at a level of 6 mL (which corresponds to 2-MNQ concentration of 123.6 µg/mL), while 2-MNQ applied alone affected white mustard and radish seedlings root elongation at lower concentrations—white mustard at 5 µg/mL and radish at 20 µg/mL. This can be explained by the fact that *I. glandulifera* leaf extract, except 2-MNQ, contains some other phytochemicals such as polyphenols that have a protective (i.e., antioxidative) effect and can ameliorate 2-MNQ negative effect on germination [22]. There are only a few studies testing the impact of 2-MNQ on germination. Bieberich et al. [1] germinated seeds and juveniles’ plants (in the first-year growth) of *F. ulmaria*, *G. urbanum*, *U. dioica*, *S. fragilis* and *L. sativum* exposed to 2-MNQ. In experiments on germinated seeds, 2-MNQ reduced the root length of *U. dioica* and *L. sativum* seedlings. Although 2-MNQ inhibited growth of all juveniles’ target plants, only biomass of juveniles *U. dioica* was significantly reduced by 2-MNQ. This confirms that except species susceptibility, plant’s life stage also affects plant’s response to 2-MNQ.

Oxidative stress is complex chemical and physiological phenomenon that accompanies virtually all biotic and abiotic stresses in higher plants [10,11,23,24,25]. Oxidative stress is a result of the overproduction and accumulation of reactive oxygen species that can attack all major plant cells’ macromolecules, resulting in their damage. Plants are constantly exposed to biotic and abiotic stresses that can negatively affect plant growth. However, to defend from oxidative stress, plants developed antioxidative defense [11,23,24,25].

Therefore, in white mustard and radish seedlings exposed to *I. glandulifera* leaf extract or 2-MNQ parameters of oxidative stress were assessed. In white mustard seedlings, *I. glandulifera* leaf extract activated synthesis of antioxidants observed as increased levels of GSH and TP. In addition, an increase in oxidative damage to proteins (white mustard and radish seedlings) and oxidative damage to lipids (white mustard seedlings) were recorded. This implies that *I. glandulifera* can initiate oxidative stress in target plants. As observed in germination experiments, *I. glandulifera* extract in radish seedlings induced oxidative stress to lesser extent than in white mustard seedlings confirming that radish is less susceptible to *I. glandulifera* negative effect. Induction of oxidative stress by *I. glandulifera* is connected to growth inhibition, since strong negative correlations among oxidative stress parameters and germination parameters in white mustard and in radish seedlings were observed.

Like *I. glandulifera* leaf extract, 2-MNQ in white mustard and radish seedlings induced oxidative stress. 2-MNQ in white mustard and radish seedlings activated antioxidative defense evident by increased synthesis of GSH, SOD and TP. Also, an increase in oxidative damage to lipids and proteins was recorded. Similarly to previous experiments, 2-MNQ alone induced changes in oxidative stress parameters at lower concentrations in comparison to its concentration in *I. glandulifera* leaf extract. In addition, the activation of antioxidative defense and oxidative damage in white mustard seedlings were observed at lower 2-MNQ concentrations in comparison to radish seedlings. Strong negative correlation among oxidative stress parameters and germination parameters confirmed involvement of oxidative stress in white mustard and in radish seedlings growth inhibition.

To our knowledge, there is no study investigating oxidative stress as mechanism of action of *I. glandulifera* extract or 2-MNQ on target plants. Segura-Aguilar et al. [26] investigated effect of juglone (5-OH-1,4-naphthoquinone, NQ produced by *Juglans nigra*) on *Picea abies*. Juglone inhibited germination of *Picea abies* and induced oxidative damage to lipids (evident by increased of MDA) while no change in antioxidative defense (SOD, catalase, GST) was observed. In another study in which the impact of 2-HNQ was tested on maize coleoptile cells, 2-HNQ reduced the growth rate of coleoptile segments and increased catalase activity and level of MDA [27]. The induction of oxidative stress in mentioned studies and in our study can be explained by NQs specific structural properties; due to their specific structural properties NQs can produce reactive oxygen species [27,28]. Thus, they can easily induce activation of antioxidative defense and induction of oxidative damage to lipids and proteins, as observed in our study.

This study confirmed that 2-MNQ is main allelochemical of *I. glandulifera* since both *I. glandulifera* extract and 2-MNQ reduced growth and induced oxidative stress in tested plants in a similar manner. Based on the obtained results, the negative effects to target plants caused by *I. glandulifera* and 2-MNQ were accomplished through oxidative stress. However, the effect of *I. glandulifera* and 2-MNQ is species-dependent, i.e., species differently respond to *I. glandulifera* and 2-MNQ.

## 4. Materials and Methods

### 4.1. Chemicals and Standards Preparation

Naphthoquinone standards 2-HNQ (2-hidroxy-1,4-naphtoquinone, 98% purity) and 2-MNQ (2-methoxy-1,4-naphtoquinone, 98% purity), 2-thiobarbituric acid (TBA) and 5,5′-dithiobis-(2-nitrobenzoic acid) (DTNB) were procured from Sigma-Aldrich (St. Louis, MO, USA). Other chemicals were obtained from Kemika (Zagreb, Croatia). For the mobile phase, HPLC grade methanol (Kemika, Zagreb, Croatia) and MilliQ water (18.2 MΩ/cm) were used.

2-HNQ and 2-MNQ standard stock solutions (20 mg/mL) for HPLC analysis were prepared in HPLC grade methanol and their working standards in the concentration range (2–100 µg/mL) were prepared by diluting standard stock solutions with HPLC grade methanol.

For germination experiment 2-MNQ stock solution (20 mg/mL) as well as dilutions of stock solution, at a concentration range of 1–30 µg/mL, were prepared in methanol (p.a.).

### 4.2. Collection of Plant Material and Extract Preparation

Plant material was collected and prepared as previously described [9]. In brief, leaves of *I. glandulifera* were collected in continental part of Croatia (Čučerje, near the city of Zagreb; 45°53′36″ N 16°03′34″ E) during flowering season. The samples obtained were authenticated at University of Zagreb Faculty of Science.

In our previous study, decoction and ultrasonic extraction were compared and it was demonstrated that by decoction higher level of NQs can be extracted from plant material [9]. Therefore, in this study, decoction was used for extraction. Briefly, 500 mg of air-dried, grounded plant material was decocted in 10 mL of 96% ethanol (*v*/*v*) under reflux conditions for 45 min [9].

### 4.3. Quantification of 2-HNQ and 2-MNQ

Quantification of 2-HNQ and 2-MNQ was performed as previously described [9]. Analysis was conducted on HPLC (Agilent 1100, Santa Clara, CA, USA) equipped with a diode-array detector (DAD). Analytical column was RP-C18 (150 mm × 4.6 mm, particle size 5 μm; LiChrospher, Merck, Darmstadt, Germany). Mobile phase consisted of methanol (A) and 2% acetic acid (B); gradient elution: 0−10 min 25% B; 10−20 min 32% B; 20−35 min 45% B; 35−42 min 25% B, with a flow rate set to 1 mL/min. Injection volume was 20 μL. Chromatograms were recorded at 280 nm. For data collection ChemStation (ver B.03.01) for LC 3D software was used. NQs were quantified based on calibration curve prepared of respective standard (2-HNQ or 2-MNQ).

### 4.4. Germination Assay

White mustard (*Sinapis alba* L., Brassicaceae) and radish (*Raphanus sativus* L., Brassicaceae) seeds were purchased at a local seed store. Seeds of white mustard and radish readily germinate (their germination rate is greater than 90%) and are commonly used for phytotoxicity testing [13] and therefore were selected for this study. Prior to experiments, the seeds were kept in dark and dry place under room temperature. Germination experiments were conducted based on OECD guidelines [13]. First, seeds were immersed in 10% sodium hypochlorite solution for 10 min to ensure surface sterility. Seeds were then washed in d-water and dried.

#### 4.4.1. *I. glandulifera* Leaf Extract Germination Assay

Into each Petri dish, two pieces of filter paper were placed. On filter papers 1.5, 3 or 6 mL of *I. glandulifera* leaf extract prepared by decoction in methanol (as described under 4.2.) was evenly added. Afterwards, filter papers were allowed to dry out (i.e., methanol to evaporate). As result, filter papers were impregnated with the extract at level 1.5 mL, 3 mL or 6 mL, respectively. On impregnated filter papers in each Petri dish, 25 seeds were placed at around 1 cm distance. To ensure germination, filter papers in each Petri dish were moistened by adding 1 mL of d-water. Petri dishes were then covered, sealed and placed in incubator in control environment (Fitotron 600 PLH, Aralab, Portugal). In experiments, negative (d-water) and positive (0.02 M CuSO_4_) controls were included. After 3 days of exposure to *I. glandulifera* leaf extract, germination was stopped and seed germination rate was calculated as number of germinated seeds divided by total number of seeds in Petri dish. For each germinated seed, root length was measured.

After assessing germination rate and root length, seedlings of each Petri dish were weighed, then placed in separated bag, labeled and stored at −20 °C until biochemical analysis. Germination was carried out in triplicate and experiment was repeated at least two times.

#### 4.4.2. 2-MNQ Germination Assay

The same procedure was followed to test impact of 2-MNQ on germination rate of white mustard and radish seeds. 2-MNQ solutions in a concentration range of 1–30 µg/mL were prepared in methanol. Into each Petri dish, two pieces of filter paper were placed and then impregnated with 2-MNQ solutions at designated concentration. Afterwards, seeds of white mustard or radish (*n* = 25 per each Petri dish) were placed on impregnated filter papers. After placing seeds, filter papers were moistened with d-water (1 mL), each Petri dish sealed and placed in incubator in control environment. Following 3 days of exposure, germination rate and seedlings root length were determined as described above. After determining seedlings’ weight, seedlings of each Petri dish were placed in labeled separate bag and placed on −20 °C until biochemical analysis.

Germination assay was performed in triplicate and experiment was repeated at least two times.

### 4.5. Preparation of Seedlings Homogenate

For biochemical analysis seedlings (100 mg) of each replicate/Petri dish were homogenized in 0.5 mL PBS buffer and centrifuged (7000 rpm, 10 min). In supernatants, parameters of oxidative stress and level of total polyphenols (TP) were assessed.

### 4.6. Determination of Oxidative Stress Parameters

In supernatants of seedlings homogenates, as parameters of oxidative stress levels of malondialdehyde (MDA), protein carbonyls, glutathione (GSH) and activity of superoxide dismutase (SOD) were assessed. MDA was assessed using thiobarbituric assay spectrophotometrically at 532 nm [29]. The level of protein carbonyls was determined in reaction with 2,4-dinitrophenylhydrazine at 370 nm [30] and GSH was assessed in reaction with DTNB at 412 nm [31]. SOD activity was assessed by using commercial kit (Cayman Chemicals, Ann Arbor, MI, USA) according to producer instructions. Absorbances were read on a microplate reader (SpectraMax i3x i SpectraMax MiniMax 300, Molecular Devices, San Jose, CA, USA).

### 4.7. Determination of Total Polyphenols

TP level was determined using Folin–Ciocalteu’s reagent according to a slightly modified method of Juszczak et al. [32]. For the supernatant (20 µL) of homogenized seedlings, 80 µL of Folin’s reagent and 80 µL of 10% Na-carbonate were added and allowed to stand for 30 min. Absorbance was read at 700 nm against blank (d-water) on microplate reader (SpectraMax i3x i SpectraMax MiniMax 300, Molecular Devices, San Jose, CA, USA). Galic acid was used as a standard. TP level was calculated from the calibration curve.

### 4.8. Statistics

Results on levels of germination parameters, oxidative stress parameters and TP were presented as mean ± standard deviation. Difference in results on germination, oxidative stress parameters and TP between negative control (treated with d-water) and treated samples (with *I. glandulifera* leaf extract, 2-MNQ or 0.02 M CuSO_4_) were tested using one-way ANOVA followed by Duncan post hoc comparison test (STATISTICA 12.0, StatSoft, Inc., USA). The *p* < 0.05 was considered significant. Additionally, Pearson’s correlation coefficients (r) between different parameters were calculated.

## Figures and Tables

**Table 1:** Fresh weight of white mustard (*S. alba*) and radish (*R. sativus*) seedlings following 3 days of exposure to *I. glandulifera* leaf extract.

Treatment	*S. alba* Fresh Weight (g)	*R. sativus* Fresh Weight (g)
Negative control (d-water)	0.61 ± 0.03 ^a^	0.68 ± 0.07 ^a^
1.5 mL	0.42 ± 0.04 ^b^	0.64 ± 0.04 ^a^
3 mL	0.39 ± 0.05 ^bc^	0.58 ± 0.08 ^a^
6 mL	0.28 ± 0.08 ^c^	0.56 ± 0.02 ^a^
Positive control (0.02 M CuSO_4_)	0.27± 0.03 ^c^	0.35 ± 0.05 ^b^

Different letters indicate significantly different values at *p* < 0.05.

**Table 2:** Fresh weight of white mustard (*S. alba*) and radish (*R. sativus*) seedlings following 3 days of exposure to 2-methoxy-1,4-naphtoquinone (2-MNQ).

Treatment	*S. alba* Fresh Weight (g)	*R. sativus* Fresh Weight (g)
Negative control (d-water)	0.34 ± 0.04 ^a^	0.44 ± 0.03 ^a^
1 µg/mL	0.31 ± 0.03 ^a^	0.45 ± 0.02 ^a^
5 µg/mL	0.29 ± 0.02 ^ab^	0.41 ± 0.05 ^a^
10 µg/mL	0.28 ± 0.07 ^b^	0.39 ± 0.05 ^ab^
20 µg/mL	0.28 ± 0.05 ^b^	0.44 ± 0.03 ^a^
30 µg/mL	0.27 ± 0.08 ^bc^	0.43 ± 0.04 ^a^
Positive control (0.02 M CuSO_4_)	0.26± 0.06 ^bc^	0.34 ± 0.02 ^b^

Different letters indicate significantly different values at *p* < 0.05.

**Table 3:** Level of total polyphenols (TP) in seedlings of white mustard (*S. alba*) and radish (*R. sativus*) following 3 days of exposure to *I. glandulifera* leaf extract.

Treatment	*S. alba* TP Level (µg/mL)	*R. sativus* TP Level (µg/mL)
Control (d-water)	342.3 ± 21.9 ^d^	373.9 ± 21.9 ^c^
1.5 mL	350.1 ± 26.6 ^cd^	405.3 ± 22.9 ^bc^
3 mL	397.3 ± 12.7 ^c^	420.7 ± 19.7 ^bc^
6 mL	467.3 ± 18.9 ^b^	424.3 ± 24.0 ^b^
PC (0.02M CuSO_4_)	589 ± 12.1 ^a^	547.8 ± 14.9 ^a^

PC—positive control. Different letters indicate significantly different values at *p* < 0.05.

**Table 4:** Level of total polyphenols (TP) in seedlings of white mustard (*S. alba*) and radish (*R. sativus*) following 3 days of exposure to 2-methoxy-1,4-naphthoquinone (2-MNQ).

Treatment	*S. alba* TP Level (µg/mL)	*R. sativus* TP Level (µg/mL)
Control (d-water)	351 ± 15.7 ^d^	372 ± 18.0 ^c^
1 µg/mL	344.3 ± 14.8 ^d^	395 ± 12.2 ^c^
5 µg/mL	396.7 ± 10.8 ^c^	402 ± 11.5 ^c^
10 µg/mL	416 ± 9.6 ^c^	442.3 ± 9.3 ^b^
20 µg/mL	475.3 ± 10.1 ^b^	465.7 ± 21.0 ^b^
30 µg/mL	510.3± 10.5 ^a^	506 ± 7.9 ^a^
PC (0.02 M CuSO_4_)	525.7 ± 14.1 ^a^	522.7 ± 15.1 ^a^

PC—positive control. Different letters indicate significantly different values at *p* < 0.05.

## Data Availability

The data presented in the manuscript are available on request from corresponding author.

## Supplementary Materials

The following supporting information can be downloaded at: https://www.mdpi.com/article/10.3390/plants14182901/s1, Table S1. Correlation coefficients (r) of white mustard (*S. alba*) germination and oxidative stress parameters following 3-days exposure to *I. glandulifera* leaves extract. Table S2. Correlation coefficients (r) of radish (*R. sativus*) germination and oxidative stress parameters following 3-days exposure to *I. glandulifera* leaves extract. Table S3. Correlation coefficients (r) of white mustard (*S. alba*) germination and oxidative stress parameters following 3-days exposure to 2-MNQ. Table S4. Correlation coefficients (r) of radish (*R. sativus*) germination and oxidative stress parameters following 3-days exposure to 2-MNQ.
