# Supplementary material for: Mechanism of Impatiens glandulifera Royle Allelopathy to Sinapis alba L. and Raphanus sativus L. Germination Is Through Oxidative Stress
Source: Plants (Basel). 2025 Sep 18;14(18):2901. doi: 10.3390/plants14182901 (PMC12473229; doi:10.3390/plants14182901)
Supplement: Supplementary file 1 [file plants-14-02901-s001.zip › plants-3823191-supplementary.pdf]

Suppl. Table S1. Correlation coefficients (r) of white mustard (*S. alba*) germination and oxidative stress parameters following 3-days exposure to *I. glandulifera* leaves extract

	Germination	Root length	Fresh weight	GSH	SOD	MDA	Carbonyls	Polyphenols
Germination	1.000	0.884	0.787	-0.926	-0.902	-0.968	-0.904	-0.968
Root length		1.000	0.760	-0.833	-0.803	-0.872	-0.788	-0.878
Fresh weight			1.000	-0.730	-0.621	-0.662	-0.852	-0.769
GSH				1.000	0.878	0.924	0.822	0.894
SOD					1.000	0.957	0.694	0.877
MDA						1.000	0.826	0.947
Carbonyls							1.000	0.883
Polyphenols								1.000

Suppl. Table S2. Correlation coefficients (r) of radish (*R. sativus*) germination and oxidative stress parameters following 3-days exposure to *I. glandulifera* leaves extract

	Germination	Root length	Fresh weight	GSH	SOD	MDA	Carbonyls	Polyphenols
Germination	1.000	0.800	0.923	-0.967	-0.973	-0.992	-0.855	-0.896
Root length		1.000	0.764	-0.685	-0.830	-0.806	-0.851	-0.891
Fresh weight			1.000	-0.932	-0.938	-0.903	-0.890	-0.887
GSH				1.000	0.949	0.955	0.797	0.835
SOD					1.000	0.973	0.882	0.930
MDA						1.000	0.836	0.882
Carbonyls							1.000	0.911
Polyphenols								1.000

Suppl. Table S3. Correlation coefficients (r) of white mustard (*S. alba*) germination and oxidative stress parameters following 3-days exposure to 2-MNQ

	Germination	Root length	Fresh weight	GSH	SOD	MDA	Carbonyls	Polyphenols
Germination	1.000	0.884	0.730	-0.918	-0.944	-0.922	-0.884	-0.959
Root length		1.000	0.766	-0.858	-0.944	-0.838	-0.796	-0.876
Fresh weight			1.000	-0.794	-0.755	-0.744	-0.763	-0.831
GSH				1.000	0.914	0.884	0.850	0.899
SOD					1.000	0.888	0.828	0.949
MDA						1.000	0.857	0.859
Carbonyls							1.000	0.841
Polyphenols								1.000

Suppl. Table S4. Correlation coefficients (r) of radish (*R. sativus*) germination and oxidative stress parameters following 3-days exposure to 2-MNQ

	Germination	Root length	Fresh weight	GSH	SOD	MDA	Carbonyls	Polyphenols
Germination	1.000	0.789	0.746	-0.914	-0.892	-0.876	-0.862	-0.806
Root length		1.000	0.739	-0.890	-0.822	-0.792	-0.722	-0.759
Fresh weight			1.000	-0.738	-0.721	-0.756	-0.714	-0.739
GSH				1.000	0.905	0.931	0.958	0.862
SOD					1.000	0.886	0.916	0.898
MDA						1.000	0.842	0.774
Carbonyls							1.000	0.916
Polyphenols								1.000
